# Effect of Low Molecular Weight Oligopeptides Isolated from Sea Cucumber on Diabetic Wound Healing in db/db Mice

**DOI:** 10.3390/md16010016

**Published:** 2018-01-08

**Authors:** Di Li, Lin Li, Teng Xu, Tianxing Wang, Jinwei Ren, Xinran Liu, Yong Li

**Affiliations:** 1 Department of Nutrition and Food Hygiene, School of Public Health, Peking University, Beijing 100191, China; lidiyy@126.com (D.L.); lynn1992lilin@126.com (L.L.); luolyusheng@bjmu.edu.cn (T.X.); wtx5222@sohu.com (T.W.); ren_jinwei@126.com (J.R.); liuhappy07@163.com (X.L.); 2 Medical Center, SinoMed Peptide Valley Bioengineering Co., Ltd., Beijing 100027, China

**Keywords:** sea cucumber, oligopeptide, diabetes mellitus, wound healing

## Abstract

Impaired wound healing is a major clinical problem in patients with diabetes and is the leading cause of lower limb amputation. This study is aimed to observe the effects of small molecule oligopeptides isolated from sea cucumber (SCCOPs) on the wound healing process in diabetic mice. Ninety db/db male mice were divided into five groups, including the model control group, whey protein group (0.50 g/kg) and three SCCOPs dose groups (0.25 g/kg, 0.50 g/kg and 1.00 g/kg). Additionally, 18 db/m male mice were used as normal control group. After full-thickness incisions on the dorsum, mice in SCCOPs-treated groups were intragastrically administered SCCOPs, while others were administered vehicle or whey protein. Mice were sacrificed on days 4, 7 and 14. The wound healing condition, inflammatory response, angiogenesis, collagen deposition, oxidative stress and nutritional status were evaluated. A pathological report showed increased vascularisation, collagen deposition and epithelialisation in SCCOPs-treated groups. SCCOPs-treated mice showed decreased C-reactive protein (CRP), interleukin (IL)-6, IL-8, tumor necrosis factor (TNF)-α, chemokine (C-C motif) ligand 2 (CCL2) and reactive oxygen species (ROS) contents, and increased IL-10, stromal cell-derived factor-1 alpha (SDF-1α), nitric oxide (NO), albumin (ALB), prealbumin (PA) and transferrin (TRF) levels and vascular endothelial growth factor (VEGF) expression. All parameters were significant (*p* < 0.05) in comparison to model control group. These results suggest that treatment with SCCOPs can promote significant wound healing in diabetic mice.

## 1. Introduction

Diabetes is a public health problem worldwide. Studies showed that the number of diabetics in the world was 415 million in 2015, and it may reach 642 million by 2040 [1]. The complications caused by diabetes are the main cause of disability and death in diabetic patients, among which impaired wound healing is a major clinical problem and is the leading cause of lower extremity amputation [2,3,4]. Thus, care for diabetic wounds remains a significant clinical problem and the development of therapies to improve wound healing in diabetic patients is of critical importance. Currently, pharmacologic therapies tried to address this issue through either the application of labgrown dermal substitutes, stem cell therapies, or the delivery of super-physiological concentrations of recombinant growth factors [5]. However, these have proven to be difficult to maintain, and have led in some cases to serious undesired side effects [6]. Thus, compounds that provide greater symptomatic relief with less overall toxicity and minimal risks are preferred.

In recent years, nutritional interventions are receiving increasing attention, and there have been numerous studies showing that natural food ingredients are effective in immune responses, anti-inflammation and resistance to diabetes [7,8,9]. Up to now, a wide variety of bioactive peptides has been isolated from animals, plants and microorganisms. They were demonstrated to have a wide range of physiological functions, such as antimicrobial, cholesterol-lowering, antioxidant, collagen synthesis-improvement or immunomodulatory activities [10,11]. Bioactive peptides have great potential in medical and health care areas. The statistics showed that peptide therapeutics shared 17.5 billion US dollars in the global market in 2015, and the share was predicted to reach 47 billion US dollars by 2025 [12]. As of February 2016, the Food and Drug Administration (FDA) has approved more than 60 kinds of peptide drugs [13], and over 400 kinds of peptide therapeutics are in the pre-clinical or clinical trial stage [12].

As marine organisms comprise approximately one-half of the total global biodiversity, the sea is an enormous resource for novel compounds [14]. Sea cucumber, used as a restorative medicine, has been widely used in Asia for thousands of years. The detailed medical applications of sea cucumber were officially recorded in the “Compendium of Materia Medica (Bencao Gangmu)” in 1758, where it was held in as high esteem as ginseng. The dried body wall of sea cucumber contains many biologically active substances and nutrients, such as about 90% of protein (polypeptides), 6% of polysaccharides (oligosaccharides) and 4% of lipids in organics [15]. It also contains multivitamins (vitamin A, B1, B2 and B3) and minerals (especially calcium, magnesium, iron and zinc) [16]. Such composition makes sea cucumbers potential candidates for clinical nutrition. In addition, sea cucumbers may have many beneficial biological activities, including anticancer, antioxidant, anti-inflammatory, antithrombotic, antitumor, antimicrobial, immunomodulatory activities, and improving wound healing [16,17]. Small molecule oligopeptides isolated from sea cucumber (SCCOPs), as short chain polypeptides, are the optimal and easily absorbable nitrogen source and have been demonstrated to possess different pharmacological activities like antioxidant, anti-inflammatory, anti-aging, immunomodulatory, angiogenesis-increasing, etc. [18], but their effects on improving wound healing are rarely reported. Our previous studies showed that oligopeptides isolated from chum salmon had a beneficial effect on diabetic wound healing [14]. Accordingly, we speculate that SCCOPs have the ability to improve diabetic wound healing. Thus, in the present study, we investigated whether SCCOPs could improve wound healing in diabetic mice (db/db) and its possible mechanisms.

## 2. Results

### 2.1. General Information

As shown in Table 1, the body weight in diabetes groups (model control group, 0.50 g/kg whey protein group and SCCOPs-treated groups) was significantly higher than that of in normal control group, while no differences were observed among MC, WP and SCCOPs-treated groups over the process of the experiment. Blood glucose levels in diabetic mice used in the present study were consistently higher than 300 mg/dL and the levels were not changed by administration of SCCOPs.

**Figure 1:**
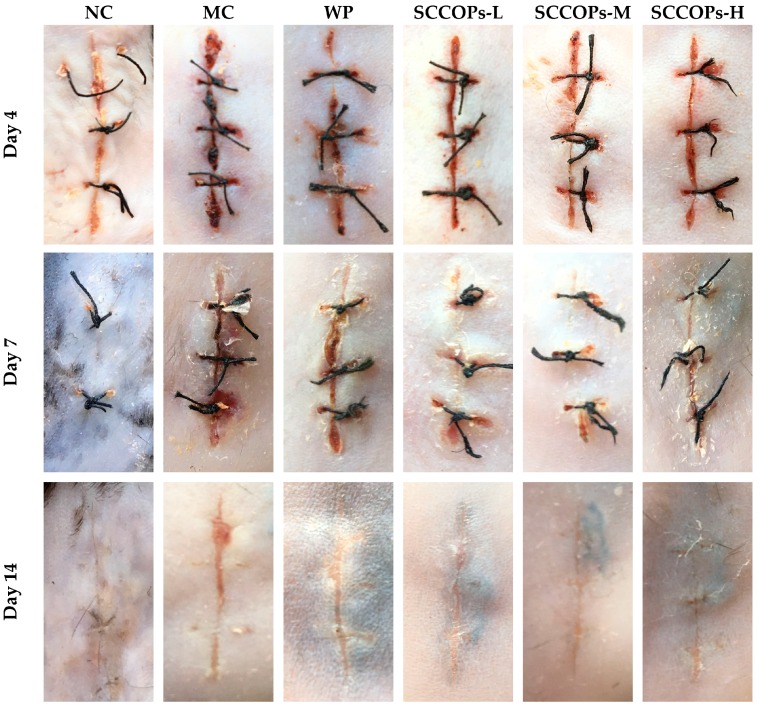
Representative macroscopic images of wounds in mice on days 4, 7 and 14. (SCCOPs, small molecule oligopeptides isolated from sea cucumber; NC, normal control group; MC, model control group; WP, 0.50 g/kg whey protein group; SCCOPs-L, 0.25 g/kg SCCOPs group; SCCOPs-M, 0.50 g/kg SCCOPs group; SCCOPs-H, 1.00 g/kg SCCOPs group).

As illustrated in Figure 1, diabetic mice (MC, WP and SCCOPs groups) showed significantly delayed wound healing when compared with non-diabetic mice (NC). In addition, in comparison with MC, the wound site in SCCOPs-treated groups seemed less bruised and swollen on day 4; on day 7, the wound site in SCCOPs-treated groups was no longer bruised and swollen, and the incision tended to close; on day 14, the incision in SCCOPs-treated groups closed more tightly, and the surface of the wound became smoother. Moreover, the wound status in SCCOPs-treated groups was better than that of in WP over the process of the experiment.

### 2.2. Histological Analysis

**Figure 2:**
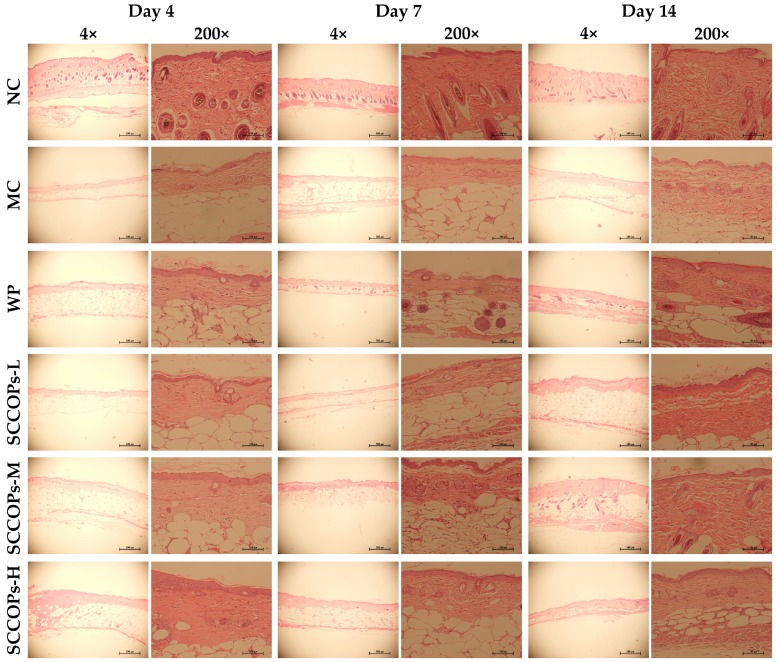
Representative micrograph of haematoxylin and eosin-stained section of wounds in mice on days 4, 7 and 14. The incision site in MC showed irregular arrangement of collagen bundles were loosely packed, and wounds demonstrated decreased vascularisation and epithelialisation. The site of wounds in SCCOPs-treated mice showed more densely packed and compactly arranged collagen bundles with increased blood vessel formation and on-going epithelialisation, reflecting a more rapidly healing wound than that of MC. (Magnification, 40× and 200×; SCCOPs, small molecule oligopeptides isolated from sea cucumber; NC, normal control group; MC, model control group; WP, 0.50 g/kg whey protein group; SCCOPs-L, 0.25 g/kg SCCOPs group; SCCOPs-M, 0.50 g/kg SCCOPs group; SCCOPs-H, 1.00 g/kg SCCOPs group).

The histological evaluation, including collagen deposition, vascularisation and epithelialisation in the incision wound area, is shown in Figure 2. Compared with MC and WP, mice in the SCCOPs-treated groups tended to demonstrate increased vascularisation, collagen deposition and epithelialisation at four days post-wounding. Vascularization was significantly higher in the SCCOPs-treated groups than in MC and WP at seven days. Furthermore, significantly abundant collagen deposition as well as denser distribution was observed in the SCCOPs-treated groups at seven and 14 days post-wounding. Moreover, epithelialization was significantly greater in the SCCOPs-treated groups than in MC and WP on day 14.

### 2.3. Evaluation of Inflammatory Response

**Figure 3:**
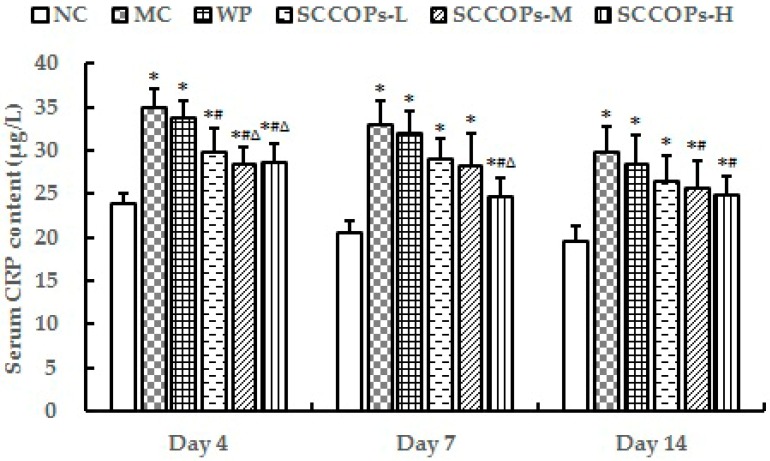
Serum CRP content in mice on days 4, 7 and 14. (CRP, C-reactive protein; SCCOPs, small molecule oligopeptides isolated from sea cucumber; NC, normal control group; MC, model control group; WP, 0.50 g/kg whey protein group; SCCOPs-L, 0.25 g/kg SCCOPs group; SCCOPs-M, 0.50 g/kg SCCOPs group; SCCOPs-H, 1.00 g/kg SCCOPs group). Values are presented as mean ± SD. * *p* < 0.05 versus NC, # *p* < 0.05 versus MC, ∆ *p* < 0.05 versus WP.

**Figure 4:**
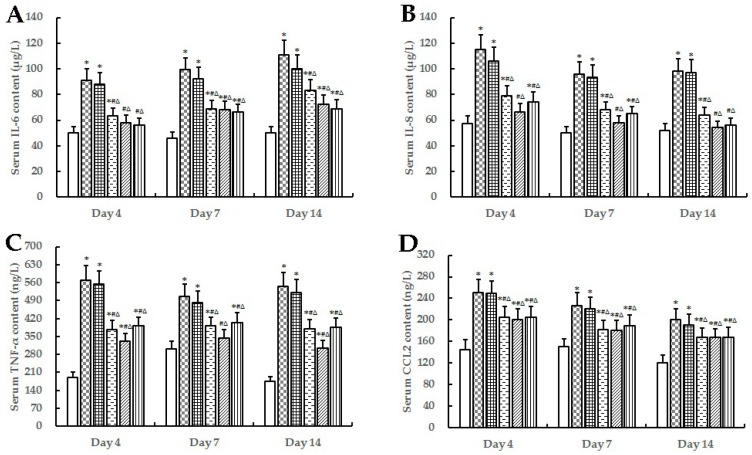

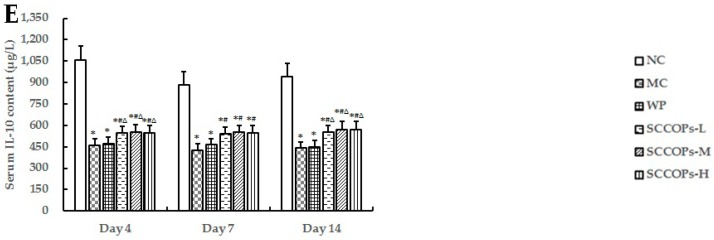
Serum IL-6 (**A**), IL-8 (**B**), TNF-α (**C**), CCL2 (**D**) and IL-10 (**E**) content in mice on days 4, 7 and 14. (IL-6, interleukin-6; IL-8, interleukin-8; TNF-α, tumor necrosis factor-α; CCL2, chemokine (C-C motif) ligand 2; IL-10, interleukin-10; SCCOPs, small molecule oligopeptides isolated from sea cucumber; NC, normal control group; MC, model control group; WP, 0.50 g/kg whey protein group; SCCOPs-L, 0.25 g/kg SCCOPs group; SCCOPs-M, 0.50 g/kg SCCOPs group; SCCOPs-H, 1.00 g/kg SCCOPs group). Values are presented as mean ± SD. Values are presented as mean ± SD. * *p* < 0.05 versus NC, # *p* < 0.05 versus MC, ∆ *p* < 0.05 versus WP.

The levels of C-reactive protein (CRP), interleukin (IL)-6, IL-8, tumor necrosis factor (TNF)-α, chemokine (C-C motif) ligand 2 (CCL2) and IL-10 in serum from non-diabetic and diabetic animals were measured as indicators of inflammatory activity (Figure 3 and Figure 4). Compared with NC, the levels of serum inflammation indexes like CRP, IL6, IL8, TNF-α, and CCL2 were significantly increased (*p* < 0.05), while IL10 levels were significantly decreased (*p* < 0.05) in diabetes groups (MC, WP and SCCOPs-treated groups). In comparison with MC and WP, the levels of CRP, IL6, IL8, TNF-α, and CCL2 were significantly decreased (*p* < 0.05), while IL10 levels were significantly increased (*p* < 0.05) in SCCOPs-treated groups.

### 2.4. Evaluation of Stromal Cell-Derived Factor-1 Alpha (SDF-1α) and NO in Serum

**Figure 5:**
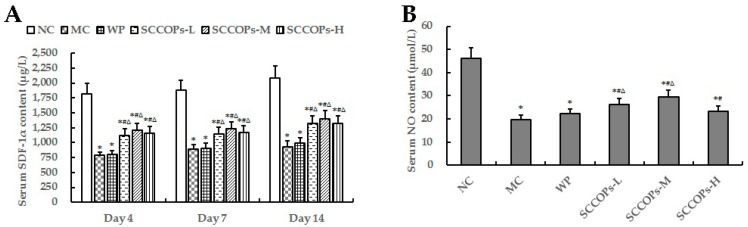
Levels of serum SDF-1α (**A**) on day 4, 7, 14, and serum NO content (**B**) on day 14 in mice. (SDF-1α, stromal cell-derived factor-1 alpha; SCCOPs, small molecule oligopeptides isolated from sea cucumber; NC, normal control group; MC, model control group; WP, 0.50 g/kg whey protein group; SCCOPs-L, 0.25 g/kg SCCOPs group; SCCOPs-M, 0.50 g/kg SCCOPs group; SCCOPs-H, 1.00 g/kg SCCOPs group). Values are presented as mean ± SD. Values are presented as mean ± SD. * *p* < 0.05 versus NC, # *p* < 0.05 versus MC, ∆ *p* < 0.05 versus WP.

The levels of serum SDF-1α on days 4, 7, 14, and serum nitric oxide (NO)content on day 14 from non-diabetic and diabetic animals were measured as indicators of vascular endothelial function (Figure 5). Compared with NC, the levels of SDF-1α and NO were found to decrease significantly (*p* < 0.05) in diabetes groups (MC, WP and SCCOPs-treated groups). In comparison with MC and WP, the levels of SDF-1α and NO were significantly increased (*p* < 0.05) in SCCOPs-treated groups.

### 2.5. Evaluation of Immunostaining for Vascular Endothelial Growth Factor (VEGF)

**Figure 6:**
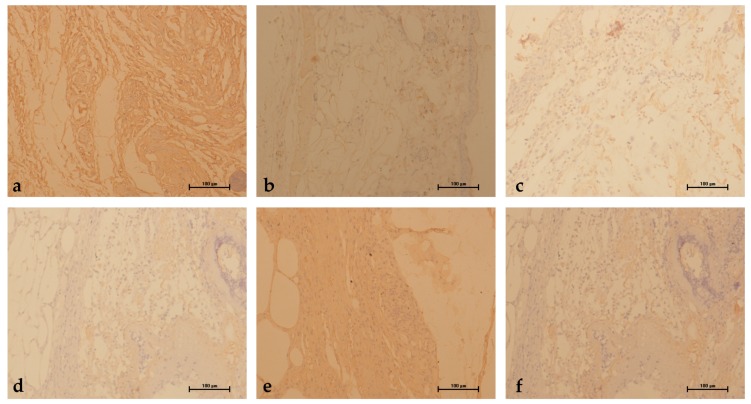
Expression of VEGF in wound tissue on day 14 after wounding in (**a**) NC; (**b**) MC; (**c**) WP; (**d**) SCCOPs-L; (**e**) SCCOPs-M and (**f**) SCCOPs-H. The VEGF immunolabeling was striking in SCCOPs-treated groups, whereas it was faint in MC and WP. (Magnification, 200×; VEGF, vascular endothelial growth factor; SCCOPs, small molecule oligopeptides isolated from sea cucumber; NC, normal control group; MC, model control group; WP, 0.50 g/kg whey protein group; SCCOPs-L, 0.25 g/kg SCCOPs group; SCCOPs-M, 0.50 g/kg SCCOPs group; SCCOPs-H, 1.00 g/kg SCCOPs group).

The VEGF immunoreactivity of wounds was examined in mice at 14 days post-wounding. As shown in Figure 6, VEGF immunolabeling was striking in SCCOPs-treated groups, whereas it was faint in MC and WP.

### 2.6. Tensile Strength

**Figure 7:**
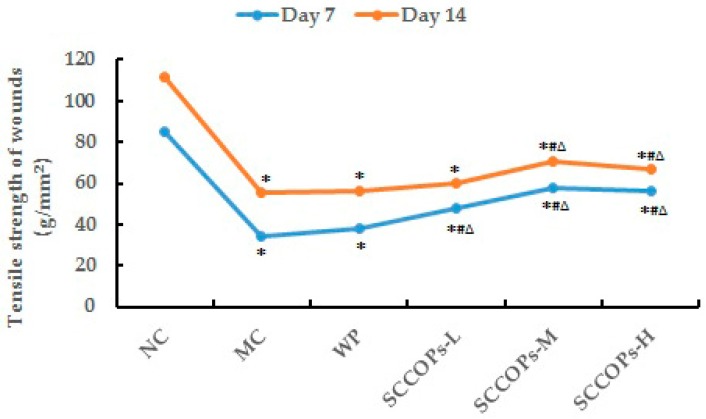
Levels of wound tensile strength on days 7 and 14 in mice. (SCCOPs, small molecule oligopeptides isolated from sea cucumber; NC, normal control group; MC, model control group; WP, 0.50 g/kg whey protein group; SCCOPs-L, 0.25 g/kg SCCOPs group; SCCOPs-M, 0.50 g/kg SCCOPs group; SCCOPs-H, 1.00 g/kg SCCOPs group). Values are presented as mean ± SD. Values are presented as mean ± SD. * *p* < 0.05 versus NC, # *p* < 0.05 versus MC, ∆ *p* < 0.05 versus WP.

The tensile strength is the strength of a healing wound and is measured experimentally by the amount of force required to disrupt it. In the beginning, a wound will have little breaking strength because the clot alone will be holding the edges together. The results of wound tensile strength measurement at seven and 14 days indicate that a lower wound tensile strength was measured in diabetes groups (MC, WP and SCCOPs-treated groups) (*p* < 0.05). Compared with MC and WP, a higher wound tensile strength was measured in SCCOPs-treated groups (*p* < 0.05). The differences among groups were statistically significant (Figure 7).

### 2.7. Collagen Accumulation

**Figure 8:**
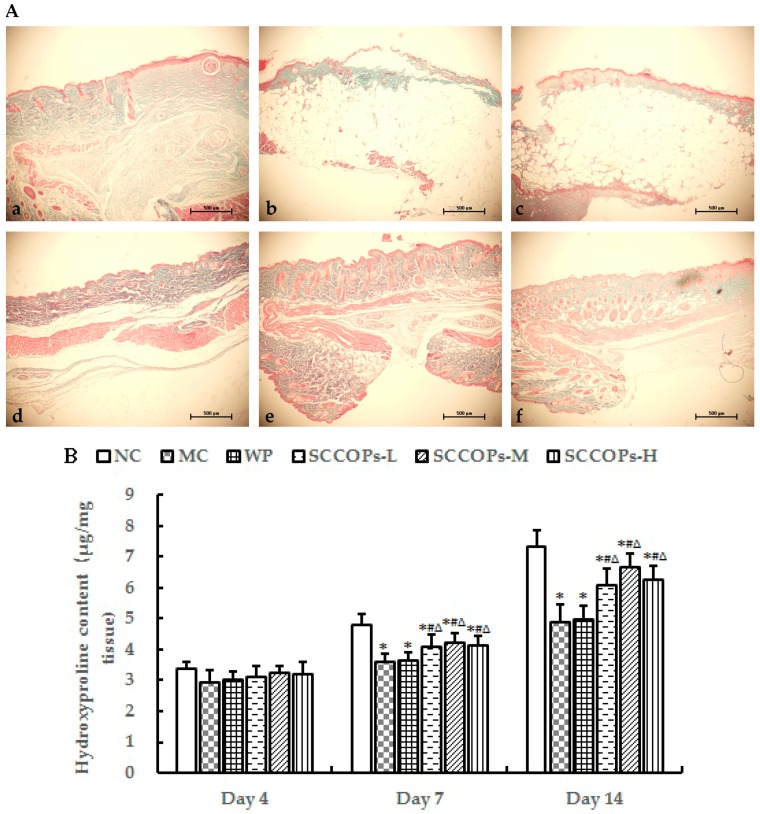
Collagen accumulation in wound areas in mice. (**A**) representative high power view light micrographs (Masson’s trichrome staining) in (**a**) NC, (**b**) MC, (**c**) WP, (**d**) SCCOPs-L, (**e**) SCCOPs-M and (**f**) SCCOPs-H on day 14 (Magnification, 200×); (**B**) hydroxyproline levels in the incision wound tissue in mice on days 4, 7 and 14. (SCCOPs, small molecule oligopeptides isolated from sea cucumber; NC, normal control group; MC, model control group; WP, 0.50 g/kg whey protein group; SCCOPs-L, 0.25 g/kg SCCOPs group; SCCOPs-M, 0.50 g/kg SCCOPs group; SCCOPs-H, 1.00 g/kg SCCOPs group). Values are presented as mean ± SD. Values are presented as mean ± SD. * *p* < 0.05 versus NC, # *p* < 0.05 versus MC, ∆ *p* < 0.05 versus WP.

Collagen deposition plays an important role in granulation tissue formation. Compared with NC, the wound revealed a marked and robust decrease in the organization of collagen fibers bridging the gaps in diabetes groups (MC, WP and SCCOPs-treated groups). In addition, collagen fibers were more organized and dense in SCCOPs-treated groups than in MC and WP (Figure 8A). To confirm the histological observations, hydroxyproline levels were measured in the lesions on days 4, 7 and 14 after wounding (shown in Figure 8B). The results showed no significant differences among groups on day 4 (*p* > 0.05). The hydroxyproline concentration increased significantly in SCCOPs-treated groups on days 7 and 14 in comparison with MC and WP (*p* < 0.05).

### 2.8. Evaluation of Reactive Oxygen Species (ROS) in Serum

**Figure 9:**
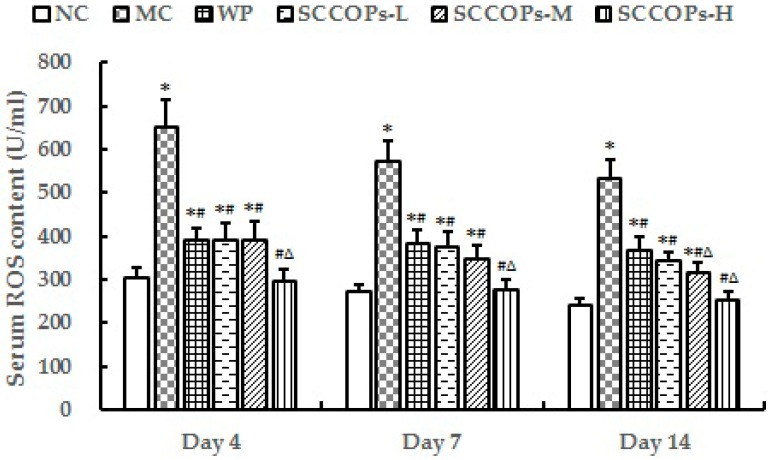
Serum ROS levels in mice on days 4, 7 and 14. (ROS, reactive oxygen species; SCCOPs, small molecule oligopeptides isolated from sea cucumber; NC, normal control group; MC, model control group; WP, 0.50 g/kg whey protein group; SCCOPs-L, 0.25 g/kg SCCOPs group; SCCOPs-M, 0.50 g/kg SCCOPs group; SCCOPs-H, 1.00 g/kg SCCOPs group). Values are presented as mean ± SD. Values are presented as mean ± SD. * *p* < 0.05 versus NC, # *p* < 0.05 versus MC, ∆ *p* < 0.05 versus WP.

The level of reactive oxygen species (ROS) in serum from non-diabetic and diabetic animals was measured as an indicator of oxidative stress. Compared with NC, the levels of ROS were found to increase significantly in diabetes groups (MC, WP and SCCOPs-treated groups) (*p* < 0.05). In comparison with MC and WP, the levels of ROS were significantly decreased in SCCOPs-treated groups (*p* < 0.05) (Figure 9).

### 2.9. Albumin, Prealbumin and Transferrin Concentrations in Serum

The levels of serum albumin (ALB), prealbumin (PA) and transferrin (TRF) were measured experimentally (Table 2). Compared with NC, the levels of ALB, PA and TRF were significantly lower in diabetes groups (MC, WP and SCCOPs-treated groups) on days 4, 7 and 14 (*p* < 0.05). Compared with MC, ALB levels were significantly increased on day 7 in SCCOPs-L, SCCOPs-M and SCCOPs-H, and significantly increased on day 14 in SCCOPs-M and SCCOPs-H (*p* < 0.05); PA levels were significantly increased on days 4 and 7 in SCCOPs-M and SCCOPs-H (*p* < 0.05), and significantly increased on day 14 in SCCOPs-L, SCCOPs-M and SCCOPs-H (*p* < 0.05); TRF levels were significantly increased on days 4, 7 and 14 in SCCOPs-L, SCCOPs-M and SCCOPs-H (*p* < 0.05). However, there were no significant differences on ALB, PA and TRF levels between WP and SCCOPs groups (*p* > 0.05).

## 3. Discussion

Derived from hydrolysis of sea cucumber, SCCOPs, whose major components are small molecule oligopeptides, have potential wound healing enhancing activity. To our knowledge, the present study is the first to show that oral administration of SCCOPs accelerates cutaneous wound healing in diabetic mice.

Wound healing is a complex process, which is often artificially compartmentalized into three phases: inflammation, proliferation, and remodeling [19]. The inability of wounds to heal in diabetes mellitus patients is associated with an abnormality in one or more phases of the healing process. Inflammation is a double-edge sword in wound healing. In normal settings, inflammatory response should occur rapidly and be sustained for 3–4 days to allow the occurrence of subsequent phases of wound healing [20,21,22]. This requires that inflammatory cells, such as neutrophils and macrophages, migrate to the wound area and phagocytize necrotic tissue and microorganisms. However, the inflammatory response in diabetesbecomes prolonged and heightened, and is recognized as a major culprit that contributes to impaired healing [23]. Both platelets and leukocytes release pro-inflammatory cytokines that provide a chemotactic gradient for additional leukocytes to enhance the inflammatory process. These include IL-6, IL-8 and TNF-α [24]. In addition, CCL2 is a pro-inflammatory chemokine to jumpstart the macrophage response and significantly promotes inflammation [23]. In addition, CRP is one of the fastest reacting acute phase proteins and therefore a useful marker for the early diagnosis of inflammation [25]. However, as an anti-inflammatory cytokine, IL-10 can promote wound healing by limiting the inflammatory response [26]. The results of the present study showed that treatment with SCCOPs significantly reduced the levels of IL-6, IL-8, TNF-α, CCL2 and CRP, whereas elevated IL-10 concentrations in serum of diabetic mice. Taken together, SCCOPs administration significantly reduced the inflammatory response.

Angiogenesis during wound repair serves the dual function of providing the nutrients required by supplying essential nutrients and oxygen to the wound site, and promoting granulation tissue formation [21,27,28]. In the present study, SCCOPs were found to increase angiogenesis in the granulation tissue of SCCOPs-treated diabetic mice as evidenced by histological evaluation. Elevated levels of SDF-1α and NO in serum, and enhanced expression of VEGF, as revealed through immunohistochemistry analysis in wound tissue of SCCOPs-treated diabetic mice, might be responsible for this property. SDF-1α has been demonstrated to have powerful chemotactic properties toward hematopoietic stem cells and progenitor cells, thereby inducing angiogenesis [29]. Mice that lack SDF-1 exhibit many defects including impaired hematopoiesis in the fetal bone marrow [30]. Additionally, SDF-1α plays a key role in the secretion of angiogenic factors [31]. Immunohistochemical staining showed that SDF-1α increased VEGF expression in normal cartilage, especially in the superficial zone [32]. VEGF provides signaling crucial to angiogenesis, promoting revascularization in wound healing. VEGF improves angiogenesis during the process of wound healing by stimulating the migration of endothelial cells through the extracellular matrix [33]. Consistent with our present finding, Galiano et al. [34] demonstrated that SDF-1α and VEGF local therapy in db/db mice enhanced neovascularization at the wound site through a stimulation of local angiogenesis, thus suggesting that SDF-1α or VEGF may be used to promote tissue repair in a wide variety of acute and chronic injuries, particularly in conditions like diabetes or aging [35]. Moreover, it has been shown that NO increases VEGF expression, which is the most potent antigenic factor during wound healing [36], thereby stimulating the formation of new blood vessels [37]. To sum up, we speculated that to elevate SDF-1α, NO and VEGF expression and then increase angiogenesis were one of the mechanisms whereby SCCOPs enhancing diabetic wound healing.

Skin contains up to 70% of collagen, which provides the tissue with tensile strength [20]. The biosynthesis and deposition of new collagens and their subsequent maturation play an important role in wound healing. Therefore, stimulating their synthesis or deposition would be beneficial for promoting wound healing. Fibroblasts are responsible for the synthesis, deposition, and remodeling of collagens. After migrating into wounds, fibroblasts initiate the synthesis of collagens. In the initial period, the wound will only have little tensile strength because the clot only holds the edges together. Thereafter, the tensile strength increases rapidly as collagen deposition increases and cross-linkages are formed between collagen fibres [38]. As an indicator of collagen deposition, the hydroxyproline concentration always has a positive correlation with tensile strength in the skin [39]. In the present study, a significant increase in tensile strength and hydroxyproline content were observed in SCCOPs-treated wound tissues, which was further supported by Masson staining.

Under diabetic condition, wound healing is impaired due to hyperglycemia induced excessive ROS derived from imbalanced antioxidant defense system including superoxide dismutase (SOD), catalase and glutathione peroxidases (GPx) [40,41]. Accordingly, antioxidants partly improve the healing in diabetic skin wounds [42]. It was demonstrated that, in some injury cases, ointments containing SOD promoted wound healing. Our result is consistent with our previous study and other studies, demonstrating that SCCOPs has potent antioxidant and free radical scavenging effects [18,24,43,44]. Hence, we speculated that SCCOPs enhanced induction of antioxidant levels at an initial stage of healing, which might be an important contributory factor in their healing property.

In addition, after surgery, the body is in a hypermetabolic state and needs a higher amino acid intake to guarantee collagen production. As a consequence, in order to exclude the confounding factors that dietary protein intake might influence wound healing, the body weight was measured. In addition, ALB, PA and TRF were chosen to reflect the nutritional status in the body. The results showed that no significant differences on body weight and levels of ALB, PA and TRF were observed among WP and SCCOPs-treated groups on days 4, 7 and 14, indicating that SCCOPs’ ability to improve diabetic wound healing may not be attributed to dietary protein intake.

## 4. Materials and Methods

### 4.1. Preparation and Identification of SCCOPs

SCCOPs were derived from fresh sea cucumber by enzymatic hydrolysis and donated by SinoMed Peptide Valley Bioengineering Co., Ltd. (Beijing, China). Oligopeptide samples were purified and determined, and the amino acid composition was analysed [18]. The content with a relative molecular mass greater than 2000 was 2%, between 1000 and 2000 was 8.56%, and less than 1000 (small molecule oligopeptides) in SCCOPs was 89.44%; the content of free amino acids amounted to 7.496%. The amino acid composition is shown in Table 3.

Basal diet (AIN-93G rodent diet) were produced by HFK Bioscience Co. Ltd. (Beijing, China). Dietary ingredients were thoroughtly mixed in a mixture, made into pellets and air-dried at room temperature.

### 4.2. Animals and Groups

A total of 90 male db/db mice and 18 male db/m mice (10–12 weeks old, 18–22 g) were obtained from Cavens Experimental Animal Co., Ltd., Changzhou, China. Mice were housed two per plastic cages with free access to chow and tap water in a Specific Pathogen Free (SPF) filter-protected air-conditioned room with controlled temperature (21–25 °C), relative air humidity (50 ± 5%), and 12-h light/dark cycles (light on 07:30–19:30). All animals were handled in accordance with the guidelines of the Principle of Laboratory Animal Care (NIH publication No. 85-23, revised 1985) of the Peking University Animal Research Committee (www.lab.pku.edu.cn).

After a 1-week acclimation period, 90 db/db mice were randomly assigned to five groups (*n* = 18): one model control group (MC), one whey protein group (WP) and 3 SCCOPs dose groups (SCCOPs-L, SCCOPs-M and SCCOPs-H), while 18 db/m mice were considered to be normal control group (NC). Mice in SCCOPs-treated groups were intragastrically administered different dose SCCOPs (0.25 g/kg, 0.50 g/kg and 1.00 g/kg), while mice in NC, MC and WP were intragastrically administered vehicle, vehicle and 0.50 g/kg whey protein, respectively (0.1 mL/10 g). Oral administration began on the wounding day and was maintained daily until sacrifice.

### 4.3. Incision Wound Healing Model

Mice were intraperitoneally anaesthetised with 4% chloral hydrate. After shaving the dorsum, two 2.0-cm long, parallel, full-thickness incisions were performed under aseptic conditions on the left and right side of each mouse’s dorsum and were closed with surgical sutures. The parted skin was kept together and stitched with black silk at 0.5 cm intervals. Surgical thread (No. 000) and a curved needle (No. 9) were used for stitching. The continuous thread on both wound edges were tightened for good closure of the wounds. The wounding day was considered as day zero. Sutures were removed on post-operative day 7. Five, five and eight animals from each group were randomly selected for the detection of indexes on post-operative days 4, 7 and 14, respectively. A blood sample was obtained from the eyeballs of the mice and then sacrificed. The serum was separated by centrifugation (3500× *g* for 10 min at 4 °C) for biochemical assays. Wound strips were removed along incision including 3 mm from the edges and were used as experimental samples. The samples from the left side were used for hydroxyproline measurement and the samples of the right side were used for tensile strength measurement and histology.

### 4.4. Measurement of Tensile Strength

Tensile strength, the resistance to breaking under tension, indicates how much the repaired tissue resists damage under tension and may indicate the quality of repaired tissue. The wounds at the back of the animals were harvested on postoperative days 7 and 14. Specimens were prepared as 0.5 cm × 1.0 cm skin islands and positioned with the incision centered on an apparatus that was connected to JZ300 force transducer working with a Biomedical Signal Acquiring Processing Systems (Beijing MicroStar Technology Development Co., Ltd., Beijing, China). The tissue was gradually stretched manually by a vernier mechanism. The breaking strength at the time of wound dehiscence was noted. After measuring the wound thickness by a micrometer, the ratio of breaking strength to the surface area of the wound (mm^2^) was noted as tensile strength.

### 4.5. Biochemical Assay

The levels of IL-6, IL-8, TNF-α, CCL2, CRP, IL-10, SDF-1α and NO in mice serum were measured by an enzyme linked immunosorbent assay (ELISA), according to the kit’s instructions. The levels of serum ALB, PA and TRF were detected by Olympus AU400 automatic biochemistry analyzer (Olympus, Tokyo, Japan). The ROS contents in serum and hydroxyproline levels in skin tissue were determined with ROS and hydroxyproline detection kits according to the manufacturer’s protocols. All detection kits were purchased from Beyotime Institute of Biotechnology (Beijing, China).

### 4.6. Histological Observation

The entire wound, including a 5 mm margin of unwounded skin, was excised down to the fascia on days 4, 7 and 14. The skin specimens were fixed in 10% buffered formalin for 24 h, followed by processing for conventional paraffin embedding. 5 μm-thick sections were mounted on glass slides, dewaxed, rehydrated to distilled water, and stained with hematoxylin and eosin (H&E) or Masson’s Trichrome, and then were studied with an Olympus IX70 inverted microscope (Olympus, Tokyo, Japan) from 40× to 200× magnification by two pathologists without knowledge of the previous treatment.

### 4.7. Immunohistochemistry

For the immunohistochemical detection of VEGF, paraffin-embedded tissues were sectioned (5 μm), deparaffinised and heated for 10 min in a microwaveoven in 10 mM sodium citrate buffer, pH 6.0. Endogenous peroxidase activity was blocked by treating sections with 0.3% hydrogen peroxide in methanol for 15 min. Tissues were treated with polyclonal rabbit anti-VEGF antibody (Santa Cruz Biotechnology, Inc., Santa Cruz, CA, USA; dilution 1:300) overnight at 4 °C. Specific labelling was detected with a peroxidaseconjugated goat anti-rabbit IgG and avidin–biotin peroxidase complex. Slides were then mounted with coverslips and analysed by two pathologists, blinded to the procedure.

### 4.8. Statistical Analysis

Statistical analyses were performed using SPSS software (version 19.0, SPSS Inc., Chicago, IL, USA). All of the data were presented as mean ± standard deviation (SD). Data from each group were statistically analyzed by one-way analysis of variance. All reported *p*-values were two-sided. A value of *p* < 0.05 was considered significant.

## 5. Conclusions

In conclusion, the present study indicated that SCCOPs might promote recovery of wounds in diabetic mice after a back-skin section. The reason for the significant wound healing activity of SCCOPs may be due to their multiple target therapy properties, including reducing inflammatory response, improving angiogenesis and collagen deposition, and antioxidation. Taken together, all evidence above makes it a new treatment concept for diabetic wounds in clinical practice. This kind of natural source for nutritional intervention may be beneficial for the treatment of diabetic wound healing. However, more studies are needed to completely understand the functions, and explore the future mechanisms of the effects of SCCOPs on the diabetic wound healing process.

## Figures and Tables

**Table 1:** Effect of oral small molecule oligopeptides isolated from sea cucumber (SCCOPs) treatment on body weight in the experimental groups.

Time	NC	MC	WP	SCCOPs-L	SCCOPs-M	SCCOPs-H
Day 0	29.42 ± 1.71	54.87 ± 3.75 *	53.48 ± 5.24 *	54.64 ± 4.62 *	55.64 ± 3.42 *	55.57 ± 5.56 *
Day 4	27.05 ± 2.04	49.44 ± 3.57 *	49.23 ± 5.31 *	49.61 ± 4.41 *	51.03 ± 3.04 *	50.29 ± 5.68 *
Day 7	28.21 ± 2.09	49.77 ± 1.54 *	47.83 ± 4.21 *	49.80 ± 5.56 *	49.19 ± 3.56 *	49.79 ± 3.46 *
Day 14	27.50 ± 1.58	50.94 ± 4.43 *	50.45 ± 4.42 *	48.03 ± 4.21 *	51.92 ± 3.50 *	48.40 ± 4.04 *

Values are presented as mean ± standard deviation (SD). * *p* < 0.05 versus normal control group. SCCOPs, small molecule oligopeptides isolated from sea cucumber; NC, normal control group; MC, model control group; WP, 0.50 g/kg whey protein group; SCCOPs-L, 0.25 g/kg SCCOPs group; SCCOPs-M, 0.50 g/kg SCCOPs group; SCCOPs-H, 1.00 g/kg SCCOPs group.

**Table 2:** Serum albumin (ALB), prealbumin (PA) and transferrin (TRF) concentrations on days 4, 7 and 14 in mice.

Group	ALB (μg/L)			PA (μg/mL)			TRF (nmol/L)		
	Day 4	Day 7	Day 14	Day 4	Day 7	Day 14	Day 4	Day 7	Day 14
NC	567.07 ± 50.97	527.70 ± 32.51	568.88 ± 56.10	58.83 ± 2.08	49.65 ± 4.13	53.45 ± 3.35	281.55 ± 21.26	306.12 ± 17.73	296.91 ± 19.30
MC	314.97 ± 16.11 *	212.46 ± 23.19 *	282.29 ± 20.40 *	29.55 ± 1.11 *	26.99 ± 0.82 *	28.01 ± 2.81 *	144.00 ± 8.85 *	138.81 ± 14.26 *	154.14 ± 10.60 *
WP	358.20 ± 5.80 *	304.02 ± 23.91 *^#^	341.04 ± 36.75 *	35.69 ± 3.43 *	29.97 ± 1.58 *	33.98 ± 2.27 *^#^	163.99 ± 9.49 *	166.76 ± 12.86 *^#^	170.91 ± 16.18 *
SCCOPs-L	331.36 ± 39.03 *	291.97 ± 39.64 *^#^	323.75 ± 23.80 *	34.38 ± 4.09 *	29.42 ± 2.99 *	33.39 ± 4.41 *^#^	174.97 ± 12.00 *^#^	179.90 ± 11.71 *^#^	183.74 ± 17.56 *^#^
SCCOPs-M	357.71 ± 29.58 *	311.09 ± 31.00 *^#^	351.37 ± 28.00 *^#^	35.99 ± 3.91 *^#^	35.27 ± 5.93 *^#^	33.42 ± 5.38 *^#^	177.39 ± 10.76 *^#^	183.65 ± 12.56 *^#^	186.01 ± 18.55 *^#^
SCCOPs-H	366.80 ± 21.03 *	336.59 ± 21.85 *^#^	341.50 ± 23.99 *^#^	37.07 ± 3.18 *^#^	36.32 ± 4.22 *^#^	33.63 ± 3.61 *^#^	185.16 ± 18.86 *^#^	189.22 ± 17.44 *^#^	190.59 ± 19.43 *^#^

Values are presented as mean ± SD. * *p* < 0.05 versus NC; ^#^ *p* < 0.05 versus MC. ALB, albumin; PA, prealbumin; TRF, transferrin; SCCOPs, small molecule oligopeptides isolated from sea cucumber; NC, normal control group; MC, model control group; WP, 0.50 g/kg whey protein group; SCCOPs-L, 0.25 g/kg SCCOPs group; SCCOPs-M, 0.50 g/kg SCCOPs group; SCCOPs-H, 1.00 g/kg SCCOPs group.

**Table 3:** Amino acid composition of SCCOPs.

Amino Acid	Amino Acid Composition of SCCOPs (g/100 g)
Asp	0.046
Glu	0.324
Ser	0.009
His	0.038
Gly	0.130
Thr	0.249
Arg	3.077
Ala	0.254
Tyr	0.779
Cys	0.033
Val	0.213
Met	0.179
Phe	0.593
Ile	0.175
Leu	0.972
Lys	0.411
Pro	0.015

SCCOPs, small molecule oligopeptides isolated from sea cucumber.
